# Impact of the COVID-19 pandemic and associated lockdown measures on the management, health, and behavior of the cystic fibrosis population in France during 2020 (MUCONFIN)

**DOI:** 10.3389/fpubh.2022.978627

**Published:** 2022-11-14

**Authors:** Nadia Oubaya, Thibaud Pombet, Celine Delestrain, Natascha Remus, Benoit Douvry, Dominique Grenet, Harriet Corvol, Guillaume Thouvenin, Virginie Prulière-Escabasse, Hakima Mounir, Dominique Argoud, Cédric Fretigne, Laurence Costes, Marie-Pierre Mackiewicz, Camille Jung, Laitissia Ahamada, Sophie Lanone, Bernard Maitre, Anne-Cécile Bégot, Ralph Epaud

**Affiliations:** ^1^Department of Public Health, AP-HP, Hôpitaux Henri-Mondor, Créteil, France; ^2^University Paris Est Créteil, INSERM, IMRB, Créteil, France; ^3^Laboratoire Interdisciplinaire de Recherche sur les Transformations des Pratiques Éducatives et des Pratiques Sociales (LIRTES)-EA7313, Université Paris-Est Créteil Val-de-Marne, Créteil, France; ^4^Faculté d'Éducation et de Formation, Institut Catholique de Paris (ICP), Paris, France; ^5^Centre Hospitalier Intercommunal de Créteil, Service de Pédiatrie Générale, Créteil, France; ^6^Centre des Maladies Respiratoires Rares (RESPIRARE^®^), CRCM, Créteil, France; ^7^Fédérations Hospitalo-Universitaires (FHU) Role of SENEscence in Chronic Diseases (SENEC), Créteil, France; ^8^Centre Hospitalier Intercommunal de Créteil, Service de Pneumologie, Créteil, France; ^9^Service de Pneumologie, CRCM-Centre de Transplantation Pulmonaire, Hôpital Foch, Suresnes, France; ^10^Assistance Publique-Hôpitaux de Paris, Hôpital Trousseau, Pediatric Pulmonary Department, Paris, France; ^11^Sorbonne Université, Institut National de la Santé et de la Recherche Médicale, Centre de Recherche Saint Antoine, Paris, France; ^12^Centre Hospitalier Intercommunal de Créteil, Service d'ORL, Créteil, France; ^13^Clinical Research Centre, Intercommunal Hospital of Créteil, Créteil, France

**Keywords:** cystic fibrosis, healthcare, COVID-19, lockdown, anxiety

## Abstract

**Background:**

Most of the studies on cystic fibrosis (CF) focused on SARS-CoV-2 prevalence and suggested a low incidence of infection in this population. We aimed to assess the impact of the pandemic and related lockdown measures implemented in May 2020 in response to the first wave of SARS-CoV-2 infection on healthcare access, health, and behavior in CF patients.

**Methods:**

A national questionnaire opened online from May 15th, 2020 to June 11^th^, 2020 was completed by 751 CF-patients, aged 14 years and over. It comprised questions about access to healthcare, anxiety and depression, smoking, alcohol, drug and psychotropic drug consumption, adherence to CF treatment, and constraints. A semi-structured comprehensive interview was performed no later than 1 month after the end of the lockdown in 16 CF-patients.

**Results:**

The mean age of the population was 28.0 [interquartile range (IQR) 20.0–37.0] years old. More than 75% of in-person consultations scheduled during the lockdown were canceled. Alternatively, 27% were postponed, and telehealth consultations were proposed and accepted in almost 40% of cases. More than 75% of the scheduled physiotherapy sessions were canceled and replaced mainly by self-drainage. Annual follow-up clinic visits were consistently postponed whereas required hospitalizations at CF centers for exacerbation were maintained in most cases. While 43.2% CF-patients had signs of anxiety, 51.0% presented symptoms of depression, both associated with increased use of psychotic medications and inversely correlated to COVID-19 prevalence. Among the lower and lower middle classes, very little medical information was obtained or requested by the patient, participation to sports or other activities was low, while excessive home confinement and isolation were more frequent. In contrast, in the upper middle and upper classes, individuals solicitated help to their CF centre, had more physical activities, and maintained contact with friends or families.

**Conclusion:**

The first lockdown in France had only minimal impact on the management care of CF-patients but was associated with increased symptoms of anxiety and depression, together with behavioral changes that varied with social class.

**Trial registration:**

NCT04463628.

## Introduction

The discovery of a new form of pneumonia in early December 2019 in Wuhan, Hubei Province, followed by the rapid spread of the virus in China and across all continents, has drastically changed the face of healthcare throughout the world (1). By March 2020, France had the second highest number of SARS-CoV-2 infections and the greatest number of deaths in Europe, which led the French authorities to initiate a strict lockdown from March 17^th^ to May 10^th^, 2020.

Cystic fibrosis (CF) is the most common autosomal recessive disease that leads to early mortality in Caucasians, and affects around 7,500 patients in France (2). Lung disease remains the major cause of morbidity and mortality in CF, with a progressive decline of lung function due to a vicious cycle of airway infections and inflammation (3). In France, the national newborn screening program, established in 2002, has been associated with the accreditation of specialized CF care centres (CRCM: Centre de Ressources et de Compétences de la Mucoviscidose). These centers are covering the entire population of patients in France based on their geographic location and are dedicated to the close follow-up of CF-patients from diagnosis to adulthood (4). The implementation of standard protocols and the centralization of services delivered by highly trained, multidisciplinary teams have contributed to prevention of the progressive deterioration of respiratory function described in the older CF literature. Despite apparent similarities in access to guideline-based care at an accredited CF Centre, evidence is conflicting regarding the potential role socioeconomic status, level of education but also racial and ethnic disparities as shown from a study in a CF centre in New York City (USA) (5, 6).

Pandemic viral illnesses are challenging for patients with pre-existing lung disease. Although it has been shown that virus pandemic such as H1N1 may cause significant morbidity in patient with CF (7, 8), the impact of SARS-CoV-2 infection on these patients was uncertain at the time of the first wave. Nevertheless, co-morbidities such as underlying respiratory problems were identified as risk factors for severe COVID-19 disease, and given the serious respiratory complications caused by viral infections (7, 9), CF-patients were expected to be at higher risk of severity. Surprisingly, the first reports in Europe suggested a lower impact overall than initially feared for these patients (6, 10). Since then, several publications have shown, probably due to a higher degree of contagiousness of the virus, that SARS-CoV-2 is not a benign disease for all people in this patient group (11). Nonetheless, it rapidly became evident that the impact of COVID-19 on CF-patients was not limited to the consequences of the infection but also resulted from the necessary measures taken to limit the spread of the disease such as isolation, quarantine, social distancing and community containment. These measures, particularly the national lockdown, affected daily life due to the closure of school, shops and others, but also affected the hospitals, which had to profoundly change their procedures and almost entirely focus on the care of COVID-19 patients. This forced transformation was at the expense of other critical functions, including the management of chronic diseases such as CF. Thus, the effects of the pandemic were far reaching and may have affected daily life, work performance, access to care, and mental fitness.

In this context, the present study more specifically addresses the impact of the COVID-19 pandemic and associated lockdown measures on the management, health, and behaviors of CF-patients during the first wave of COVID-19 in France. Our results are based on a national survey from patients over 14 years old using web questionnaires. This quantitative study was completed by a qualitative study including representative CF-patients who were interviewed by social science and humanities researchers.

## Methods

### Design and population

This is a French national multicentre cross-sectional study including both quantitative and qualitative analysis. The inclusion criteria were as follows: CF-patients with a chloride sweat test >60 mEq/L and/or 2 *CFTR* gene variants, 14 years-old and over, followed at one of the French CF reference centres (CRCM), covered by the national health insurance system, and willing to participate to the study (non-opposition from the patient if aged >18 years and non-opposition from the parents if the patient is <18 years old).

### Data collection

#### Quantitative study

CF-patients had to fill out an online questionnaire. The study was advertised with the support of all the CRCM and the patient association “Vaincre la Mucoviscidose” *via* websites and social networks such as Facebook and Twitter, as well as *via* emails sent by the CRCM. Patients could access the questionnaire using a generic link available in the announcement of the study. The questionnaire was open from May 15^th^, 2020 to June 11^th^, 2020 and comprised the following parts: (1) Sociodemographic characteristics (gender, age, education, employment, marital status), (2) access to healthcare during lockdown defined as follows: cancellation or rescheduling of consultations (with the doctor or the physiotherapist) by the healthcare professional or by the patient, cancellation by the patient of telehealth, cancellation or rescheduling by the hospital or by the patient of hospitalisations (planned or not) and change in the route of administration of antibiotics (oral administration instead of intravenous), (3) Compliance to medical treatment and airway clearance as assessed by the adherence score (12). The results were categorized as good compliance if the number of “yes” = 0, minor non-compliance for 1 or 2 “yes” and non-compliance for 2 or 3 “yes”; (4) Assessment of anxiety and depression: participants were asked to complete the 7-Item Generalized Anxiety Disorder Scale (GAD-7) (13) and the validated French version of 9-item Patient Health Questionnaire Depression Scale (PHQ-9) (14, 15). The total GAD-7 score ranges from 0 to 21, with a cut off of 10 indicating the presence of significant anxiety symptoms. GAD-7 scores of 5, 10, and 15 represent cut-off points for mild, moderate, and severe anxiety, respectively. The PHQ-9 score is composed of nine depressive symptom items listed in the Diagnostic and Statistical Manual of Mental Disorders-4th edition (DSMIV) for depression and ranges from 0 to 27 (14). PHQ-9 scores of 5, 10, 15, and 20 represent cut-off points for mild, moderate, moderately severe, and severe depression, respectively (14); (4) Assessment of quality of life using the specific CF CFQ 14 questionnaire that was adapted to the context of lockdown; (5) Knowledge and concern about COVID-19 and the declared prevalence of COVID-19 infections suspected or confirmed. “COVID-19 dangerous for me,” “COVID-19 dangerous for relatives,” constraints of barrier measures, constraints of lockdown, were assessed using a scale from 1 to 10, with 1 being “very low constraining” and 10 being “very constraining.”

#### Qualitative study

The questionnaire was complemented by a semi-structured comprehensive interview (16, 17) performed by Visio conference between the end of the lockdown and up to 1 month later. A sample of 16 CF-patients representative of the French population (with regard to age, sex and occupation) according to the data produced by the French Cystic Fibrosis Registry was selected from three centers: mixed (Créteil), pediatric (Trousseau, Paris) and adult (Foch, Suresnes). The different topics discussed during the interview focused on the first lockdown period and included the following: experience with the disease, treatment, care, accessibility to medical facilities, modalities and organization of work or schooling, lockdown conditions (with family or friends), organization of daily life, sports, and social networks. The interview ended with the collection of socio-demographic data.

### Outcomes

#### Primary outcome

The primary outcome was the reduction to healthcare access.

#### Secondary outcomes

The secondary outcomes included: (1) Compliance to treatment plans and airway clearance, (2) Assessment of anxiety and depression, (3) Assessment of quality of life, (4) Knowledge and concern about COVID-19 and the declared prevalence of COVID-19 infections suspected or confirmed, (5) The experience and social representations of the lockdown in CF-patients as assessed by qualitative qualitative methodology), (6) The impact of the lockdown on social inequality (assessed by qualitative methodology).

### Data analysis

#### Quantitative analysis

The results were reported according to the STROBE guidelines for observational studies. Sociodemographic characteristics of the population were described using numbers (percentages) for categorical variables and mean ± standard deviation (SD) or median [interquartile range (IQR)], as appropriate, for quantitative variables. For the primary outcome, the proportion of patients with a reduction in healthcare access during lockdown was described by percentage and 95% confidence interval. The other outcomes were described similarly than sociodemographic characteristics (2, 18). Associations between the primary outcome and, respectively, age, sex, occupation, and geographic area (Grand Est region vs. rest of the territory) and between age and, respectively, anxiety/depression, treatment, alcohol and smoking behaviors were assessed using Chi2 tests or Fisher's exact tests. Maps were built to represent anxiety, depression scores and concern and constraints about COVID-19; median scores were used in each geographic area. A map of the prevalence of COVID-19 in the general population in France at time of the questionnaire survey was built as a reference to visually compare scores between geographic areas, taking into account the prevalence of COVID-19 in these areas. All significance tests were two-tailed, and the threshold for statistical significance level was set to 5%. All analyses were performed with Stata software (v16.0 StataCorp. 2019. Stata Statistical Software: Release 16. College Station, TX: StataCorp LLC.) and R software (R Core Team, R Foundation for Statistical Computing, Vienna, Austria, 2020).

#### Qualitative analysis

Each interview was summarized by the interviewer. It allowed a pre-analysis of the interview with regard to working hypothesis and the conditions for carrying out the interview. Each interview was fully transcribed, and the analysis of the interviews was led by two members of the team. Interviews were subjected to content analysis, in particular to a thematic analysis 22. Characteristics, such as the occupation, were crossed with the last diploma to inform socially differentiated practices. Trends were identified according to three social classes: working or lower class (workers, employees), middle class (intermediate professions) and upper class (executives and higher intellectual professions, company directors). Students were defined by their parents' class. Individuals belonging to the middle class were differentiated based on their diploma. Thus, we created the lower-middle class (2 years post-graduation) and the upper-middle class (3 and 4 years post-graduation).

#### Sample size

Assuming 80% patients would have reduced health access, i.e., 20% with health access, 1,200 patients were needed to estimate this primary outcome with a precision of ±2%, with a two-tailed alpha risk of 5%. The inclusions were stopped after a month even though the sample size was not reached as the responses became scarce and the time period became far from the end of lockdown.

## Results

### Characteristics of the studied population

Baseline characteristics and geographic location of the study population are summarized in Table 1. Within a month, we collected 751 completed questionnaires, with 725 being exploitable (Supplementary Figure 1). The mean age of the studied population was 28.0 years-old [IQR 20.0–37.0], with a predominance of women (62.5%). Students were highly represented (28%), and among patients of working age, the private sector was the most represented (22%). Homemakers represented 8% of the CF-patients. For qualitative analysis, 16 CF-patients were solicited, and 15 were interviewed.

**Table 1:** Baseline characteristics of the study population.

	**No. (%)**
	**Total (*N* = 725)**
Age, med [IQR]	28.0 [20.0; 37.0]
Female	453 (62.5)
**Occupation**
Private sector	156 (21.7%)
National or public company	126 (17.5%)
Self-employed	26 (3.6%)
Seeking a first job	17 (2.4%)
Seeking a job (have already work)	46 (6.4%)
Retired	12 (1.7%)
Homemaker	60 (8.3%)
Student (including high school)	203 (28.2%)
Other	74 (10.3%)

### Consequences of the COVID-19 pandemic and associated lockdown measures on healthcare organization

More than 75% of consultations scheduled during the lockdown were canceled (Tables 2, 3-interview comments verbatim A5, A9). However, telehealth was alternatively proposed and accepted in almost 40% of cases, whereas 27% of scheduled consultations were postponed. Overall, 87.7% CF-patients [CI 95%: 84.1; 90.5] were offered access to consultations. The trend varied depending on the geographic location (Supplementary Table 1) [Grand Est region (64.0%) vs. rest of the territory (89.2%); *p* < 0.001] or the age of patients [under 18 (95.2%) vs. adults (86.3%); *p* = 0.05], and it did not vary depending on sex [male (90.2%) vs. female (85.7%); *p* = 0.20] or occupation [working (87.8%) vs. seeking for a job/retired (87.3%) vs. student (88.8) vs. others (84.6); *p* = 0.92]. More than 75% of the scheduled physiotherapy sessions were canceled (mostly by the physiotherapist), and only few telehealth or online programs were proposed (Table 3, A1), resulting in the use of self-drainage in a significant number of cases (Table 3, B1). While some patients indicated that they found it difficult to be away from their physiotherapists during the confinement (Table 3, B7), especially when they needed reassurance from these professionals (Table 3, B1), others indicated that they benefited from the advice given by their center physiotherapist or that they initiated new activities such as yoga or home exercise to stay active (Table 3, B2–6). As predicted, the routine annual follow-up visits were consistently canceled or postponed in most cases. In contrast, hospitalizations required for symptom exacerbation were maintained in most of the cases in the CF centres. The required intravenous administration of antibiotics was performed at home in more than 75% of patients. Although the qualitative questionnaire did not specifically include any questions regarding this specific topic, CF-patients who underwent qualitative interviews had no difficulty getting the needed prescription drugs during the lockdown period (Table 3, C6).

**Table 2:** Use of care by cystic fibrosis (CF) patients during the first lockdown period.

	**No. (%)**
Scheduled consultation in CF centre during lockdown period	414 (57.7)
Canceled by patient	20 (4.8)
Canceled by the hospital (no rescheduling or telehealth offered)	40 (9.7)
Telehealth offered by the hospital but refused by patient	4 (1.0)
Telehealth offered by the hospital and accepted by patient	165 (39.9)
Postponed with new appointment at the hospital	111 (26.8)
Maintained in-person at the hospital	102 (24.6)
Scheduled physiotherapy sessions	333 (46.5)
Canceled by patient	95 (28.5)
Canceled by the physiotherapist-no telehealth/online program offered	90 (27.0)
Online program offered by the physiotherapist and accepted by patient	7 (2.1)
Telehealth offered by the physiotherapist and accepted by patient	11 (3.3)
Telehealth or program offered by the physiotherapist and refused by patient	4 (1.20)
Postponed with new appointment at the hospital	34 (10.2)
Maintained at home or physiotherapist office	115 (34.5)
Scheduled annual review in CF centre during lockdown period	105 (14.7)
Canceled by patient	5 (4.8)
Canceled by the hospital (no rescheduling or telehealth offered)	21 (20.0)
Telehealth offered by the hospital but refused by patient	0 (0.0)
Telehealth offered by the hospital and accepted by patient	15 (14.3)
Postponed with new appointment in-person at the hospital	56 (53.3)
Maintained at the hospital	13 (12.4)
Hospitalization required during the lockdown	48 (6.7)
Replaced by day hospital	4 (8.3)
Performed in another unit (not CF centre)	7 (14.6)
Postponed	6 (12.5)
Maintained	33 (68.8)
Intravenous antibiotic required during the lockdown	66 (9.2)
Performed at home	51 (77.3)
Replaced by oral antibiotic treatment	2 (3.0)
Postponed	0 (0.00)
Performed at hospital	19 (28.8)
Hospitalization required during the lockdown	48 (6.7)
Replaced by day hospital	4 (8.3)

**Table 3:** Verbatim from 9 representative patient's interviews.

**Theme A: In relation to the medical institution**
A1: “I had called the physio's office and it actually went to voicemail and I listened to the voicemail, they said the office was going to be closed [during Lockdown]” (I7).
A2: “I did some research [on the impact of COVID-19 on cystic fibrosis], but in fact didn't find any... I went on the Internet...but I couldn't find any information, so I was disappointed” (I7).
A3: “At the beginning, I must admit that I felt a bit lost, in the sense that our doctors did not call us to tell us what we had to do, how we were going to protect ourselves, plus, at the beginning, we did not have a mask... I'm married, so, my husband, what can he do, can he go out shopping or not, can he maybe be near others or not, knowing that if he catches the virus and brings it home and I get it…how does it work finally, me, well, I was a little bit confused at first” (I9).
A4: “He told me that it was really serious, that I should be careful, that I shouldn't go out, that it was necessary for someone to go shopping for me, but afterwards, well, we didn't go into as much detail on the subject because they were really busy during that time, which I can understand, so they don't have a lot of time to devote to us, we can't stay on the phone for an hour, to get many explanations, so, well, that was okay, at first” (I9).
A5: “There were planned [consultations] during the confinement, but in the end everything was canceled, on the one hand, on my side. I didn't feel like coming to the hospital, I was afraid. The anxiety was terrible. Afterwards, I was offered telephone calls, so it was more convenient for me and for them too. It allows you to have follow-ups, so it also reassured me in that sense. And now [after deconfinement], I have face-to-face appointments again.” (I15)
A6: “I had questions, I asked them... he answered on a medical level, he was really available, any time, me, sometimes I sent emails in the middle of the night, yes because I had questions” (I6).
A7: “I went to a lot of different hospitals because I rebelled a little bit, I didn't agree at all with the care, it didn't suit me” (I6).
A8: “I decided after 2 weeks... I said stop the TV because you're going to go crazy... one never learns things from the news... I told my pulmonologist, ‘Listen to me, I'm referring to you, so if there are things you think I need to know, you have to tell me”' (I6)
A9: “And so it reassured her that I knew how to take care of myself, she reassured me about my own competence by telling me that I shouldn't worry because I was managing my own care by myself etc.” (I6)
A10: “And then, I contacted my CRCM by email saying, well, I don't understand, we haven't heard any news, so I was a bit panicked in fact, saying, ‘what's going on and what in fact are we doing?”. And so then I had my [coordinating] nurse who got in touch with me and she told me that they had decided to respond to all requests but to answer only if we had questions and not to communicate with us if in fact we didn't have any.” (I6)
A11: “here, they told me that it's here that you cancel all medical appointments and uh I didn't uh really understand because in fact, I told him, but uh you cancel all appointments, but then how were we going to do it really and at the time in fact one was not doing remote conferencing really, it was not even possible in the medical field, in fact it was always necessary to move and me, I am already nearby now and I always had to go to Foch etc. There was nothing done by remote conference, and so uh she tells me uh now we cancel here, you seem stable uh in relation to the information I was giving her...” (I6)
A12: “Exactly, um, in no case, um I don't know if the other patients of Foch um [her center], formulate it as I do, in no case do we feel abandoned by the medical staff, never, and that's important.” (I13)
A13: “It's true that I was very anxious because of my profession... how was it going to go, how was I going to manage... I had called the CRCM several times... to ask them, already before 16 March, if I should stop [to work]” (I8)
A14: “It's true that my doctors... told me that there had been a few cases of CF who had had COVID-19, that it hadn't been as severe as that... that they hadn't developed serious forms, so it's true that that reassured me” (I8).
A15: “Well, as time goes by, this question is always present because, um, knowing whether we will develop more severe symptoms quickly. It's true that it's frightening, it accentuates the anxiety” (I8)
A16: “a WhatsApp group where the doctor sent messages basically to parents and patients... with the latest information. It's always been quite reassuring, the most important feedback I got was that there were a hundred (patients) CF who had... been infected and about ten who were in, there were no deaths, as far as I know, and a dozen or so ended up in intensive care... but all the intensive care where the people for whom the coronavirus was a problem were patients who had had transplants... so we weren't told about the coronavirus as a threat specifically to cystic fibrosis” (I5).
A17: “my doctor used to tell all his patients ‘seeing the state of information on this pandemic, here's what to do, here's what not to do, and if you have a concern, here's the person to contact”' (I2).
A18: “she distributed information... it's mostly by e-mail that I receive information... how to deal vis-a-vis your work, under what conditions you yourself could stop, if in terms of work, working from home was not possible... some information on hygiene measures, masks, how you could get them” (I2).
**Theme B: From physiotherapy to physical and sports activities: In relation to the body**
B1: “I'm still very independent in this respect, so I know how to do my physiotherapy, I know how to take care of myself, I took care of myself for 2 months without any problem, but I must admit that at the end of 2 months, I still needed to see my physiotherapist again, if only for the purpose finally to reassure myself, the fact that he [her physiotherapist] comes and tells me, ‘It's OK, you've done your physiotherapy well', it's more for me, to reassure me” (I9).
B2: “What I told myself is that yoga is pretty good, it's an activity that's not hyper physical and at the same time it's based on breathing, so I said to myself that maybe it could do me some good and um then the fact of breathing well could also take away, if you have anxieties, some anxieties, finally, it can in fact calm the body and the mind. So I started from that principle, saying to myself that in any case it could be good for me.”(19)
B3: “It's all going to be fine now [interview conducted after deconfinement] with these physiotherapy sessions. But effectively, not having a physiotherapist, not being able to do the exercise rehabilitation sessions with the equipment like at the physiotherapist's, and then to have gained weight at the same time, it was a bit difficult” (I13).
B4: “Sports is physical activity, it's also a moment I spend with other people. So then [during the confinement], I wasn't moving, and I was all alone” (I1).
B5: “I contacted my physiotherapist, she gave me some tips on how to do sports in the garden, a bit anywhere. I followed her advice, it's fun at first, but you quickly let the rhythm go” (I15).
B6: “So in fact I started to do quite a lot of sports and above all to surpass myself in fact... so I did quite a lot of sports... I followed quite a few live streams in fact, from people who did, who before, did things for a fee, for example, yoga and things like that, and me, I took advantage of this, in fact, to enrich myself with a lot of things that I would have loved to do but that in fact usually either you have to travel for or pay a certain amount of money for etc... and so I decided to eat up as many things as possible that I wanted... it was brilliant” (I6).
B7: “So at the beginning, um, there we were, we went on a bit of a tour, um, all together, nibbling in front of the TV, um, there we were. So um it's true that at the beginning we um it was a bit of a mess at home. I don't go to a gym because, um, I'd have to be followed by a coach etc. And then, well, it's not cheap either, so um, so no gym, um, I walk. And um during the confinement, zero sports to be honest, zero sports and um and then um not long ago I started cycling a bit again, the stationary bike...”. (I13)
B8: “So yes, in my spare time, before the confinement, I used to do contemporary dance. Otherwise, I've always been a bit sporty, so I like running, sports, I don't stop doing sports because I love it.” (I15)
B9: “Yes, so we used to do sports um at home, whether it was with my mother or just me on my own, um and plus as the weather was good and we had a garden, it's true that it was nice so we could be outside. So yes, I…I continued at home, um and it's true that we also used small equipment such as elastics or weights and all that. So it's true that it allowed us to keep uh to keep up an activity in fact, to have classes anyway so uh no on that, it was also in the end uh finding a rhythm that helped.” (I8)
B10: “I started (sports) again with a friend who wanted to get back into it and that's it, we'd meet every other day at 4pm, we'd do sports for an hour, an hour and a half... on Skype... it wasn't anything that required going outside, we were in our respective rooms' (I5).
**Theme C: psychosocial adjustment to COVID-19 pandemic and associated lockdown measures**
C1: “Interviewee: Yes, we talk to them often, we even created a WhatsApp group so that we can keep in touch and everything, but with the confinement, as there are people who also have pathologies, we can't all see each other in fact.” (I7)
C2: “So I said to myself, frankly, what I should do is not go out because I don't really have any information, especially as they said that those who... those who have a lung disease are already at risk of it being complicated for them with the coronavirus. (I7)
C3: “I wash my hands quite a lot but [with COVID-19], I washed my hands all the time, all the time, as soon as I touched something that came from outside, like the mail, a parcel, in fact I spent my life washing my hands” (I9)
C4: “I kept telling my husband, when he went out, ‘you put your mask on, you took your mask, you took your alcohol gel'... I kept telling him, ‘don't forget to wash your hands as soon as you touch a door handle, there you go, then you use your alcohol gel.” (I9).
C5: “I say to myself, they don't understand the message, so there comes a time when you can't be in conflict all the time, you can't try to change people's lives, so I just let it go, the problem is that I think it isolates me” (I9)
C6: “I didn't want to go to the pharmacy because I said to myself, maybe there are sick people coming to get their medicine... I didn't want my husband to go either because it was tricky, so the pharmacist was very nice and very accommodating... I sent her my prescription by email and then she came to deliver my medicine to my house” (I9)
C7: “The problem is that sometimes what I reproach people for is that they manage to make me feel guilty, in fact, um, I know that my father-in-law wanted to come to the house even during the lockdown. He wanted to come to the house for a drink etc., so I told him no and I could see that he wasn't happy and that... so the problem is that it makes me feel guilty because I say to myself, um well I'm, I'm in... I'm the bad guy. I'm the bad guy, I tell him not to come, so because of me he doesn't see his son, um, so in a way it's not nice, well... that's how I experienced it anyway.” (I9).
C8: “my mother took it upon herself to be exposed to the virus (notably because she was in charge of doing the shopping for her, her daughter and her grandson), in fact, so when she returned home, potentially, we had to wait a fortnight to be sure... well, that's what my pulmonologist used to tell me, to make sure that no one got it, so in fact, each time it necessitated pushing back on when I could see my son, so it lasted a very long time, and then he (the pulmonologist) started talking to me about the fact that in fact there was no end date for knowing when I would see my son again (...) as my pulmonologist used to say, it was that you had to wait until you had the tests” to see your son again (I6)
C9: “basically, I had to do everything I had forbade myself to do during, for my part, over two and a half months” (I6).
C10: “at the pharmacy, we are in contact with a lot of sick people, so I'm going to wait for the [contamination]rate to decrease a little”(I1).
C11: “I already know that I am very vulnerable, very fragile, so I did not go out as soon as the lockdown was announced.” (I15)
C12: “No, I don't go out in public places. Ok so, I can walk my dog but I don't go out to places where I can meet people, it still scares me in any case.”(I15)
C13: “we clean more, we clean between each patient, the equipment, also each time, I change my gown” (I8).
C14: “Um, afterwards it's true that my doctors in [her center] told me that uh there had been a few cases of CF who had uh COVID-19 that it hadn't been as severe as it had been, that it was rather maintained, that it had not developed serious forms. So it's true that that reassured me, um, to be able to allow myself, um, it's true that so me, I live with my parents...” (I8)
C15: “we didn't put any more precautions than that or extreme precautions inside the house, it's just maybe that we had less contact but that's all” (I8).
C16: “I said, ‘well, it's not complicated, we all respect the lockdown... what we're going to do is have face-to-face aperitifs'. We're two houses side by side with a hedge that crosses between, so we made a plan of who's going into the courtyard of which house, and we each arrive with our table, our chairs, our bottle and our glass, and we don't share anything at all, and we limit ourselves to half an hour. So it was me who set this up, it lasted half an hour, we were very happy, it was Saturday evenings, the following Saturday, it lasted an hour, then it went on longer... and the seventy year old neighbor was very happy” (I2).
C17: “Nobody comes into my home [Laughs]... but otherwise, we go for a walk, so I go with my mask and everything, I don't touch anything, in the street I don't sit anywhere. When someone who doesn't have a mask passes by, I try to avoid them without appearing too hysterical either, but I'm really careful that... that when they talk to me, they have a mask, they have... in any case the people I agree to see are people who have good hygiene rules, who have known me since I was a child and who know what to do, wash their hands, put on a mask and everything.” (I4)

I1: LMC, F 26 yo; I2: UC, M, 52 yo; I3: LMC, 59 yo; I4: UC, F,16 yo; I5: UC, M,18 yo; I6: LMC, F, 31 yo; I7: PC, F,17 yo; I8: UMC, F, 27 yo; I9: LMC, F, 38 yo; I13: UC, F, 50 yo; I, Interview; F, female; M, male; yo, years old; PC, Popular category; LMC, Lower middle category; UMC, Upper middle category; UC, Upper category. Color code: dark gray, PC; light gray, LMC; very light gray, UMC; and white, UC.

### Health impact of the COVID-19 pandemic and associated lockdown measures

#### Concerns about COVID-19

**Figure 1:**
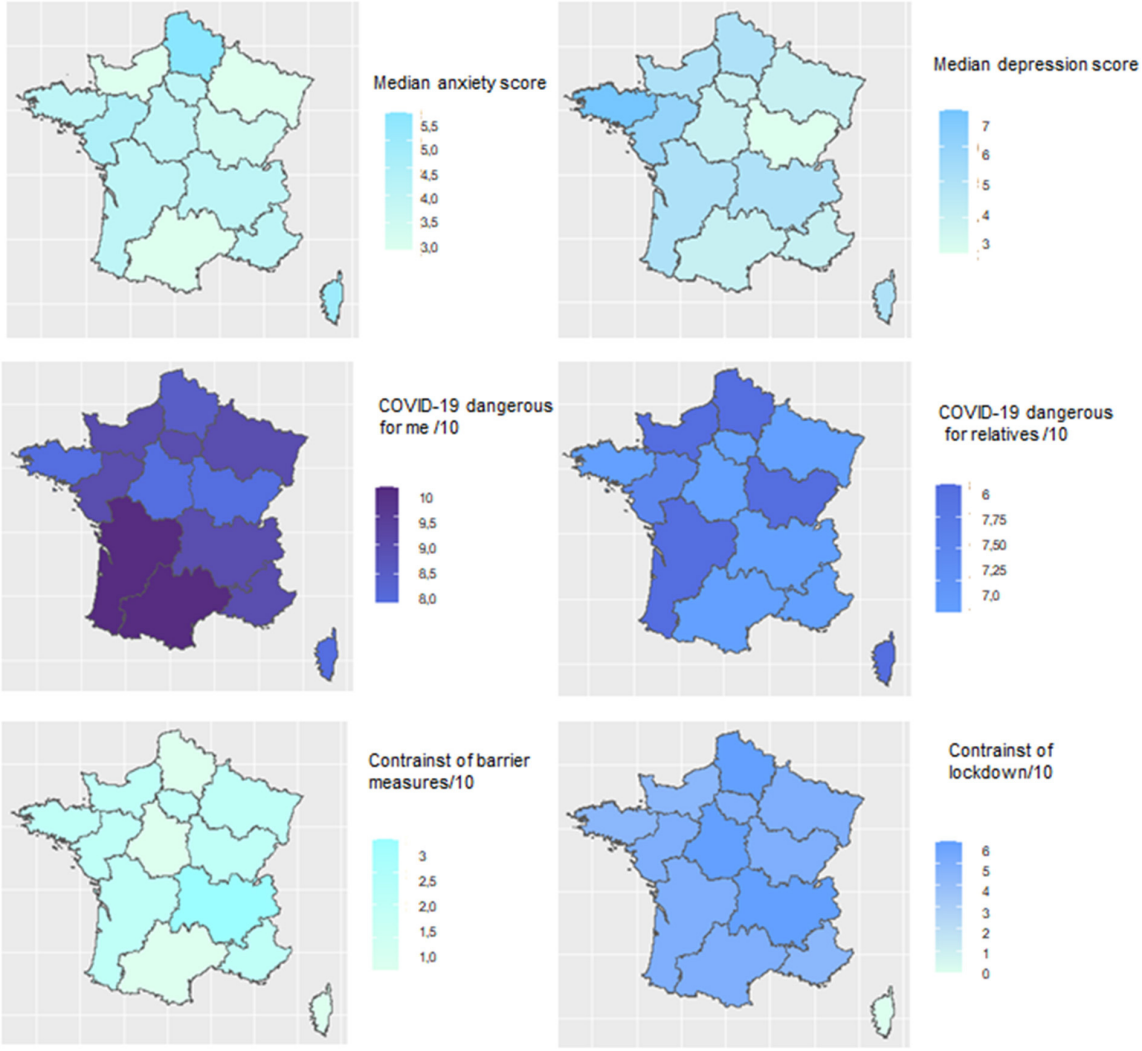
Health impact of the COVID-19 epidemic and associated lockdown measures on cystic fibrosis patients during the first wave of COVID-19 according to different geographic regions of France. Assessment of anxiety and depression: participants were asked to complete the 7-Item Generalized Anxiety Disorder Scale (GAD-7) (13) and the validated French version of the 9-item Patient Health Questionnaire Depression Scale (PHQ-9) (14, 15). The total GAD-7 score ranges from 0 to 21, with a cut off of 10 indicating the presence of significant anxiety symptoms. The PHQ-9 score is composed of nine depression symptom items listed in the Diagnostic and Statistical Manual of Mental Disorders-4th edition (DSMIV) for depression and ranges from 0 to 27 (14). The median score is indicated for each metropolitan French geographic area. “COVID-19 dangerous for me,” “COVID-19 dangerous for relatives,” constraints from barrier measures, and constraints from lockdown were assessed using a scale of 1 to 10, with 1 being “very low” and 10 being “very high.” Results are represented for each of the French geographic areas, with colors that correspond to the values in an intuitive manner (higher values are darker blue, while lower values are lighter blue).

Numerous CF-patients reported that their center initially provided insufficient information about specific risk of COVID-19 for CF-patients (Table 3, A3, A4, C9). Nevertheless, this improved overtime through interactions with physicians and coordinating nurses or through direct information from medical institutions, such as ***via*** WhatsApp groups or email diffusion (Table 3, A5–6, A8–12, A16, A18). As a result, answers to the questionnaires showed that CF-patients had significant knowledge about the SARS-CoV-2 infection. Nonetheless, 65% of CF-patients believed that they were at higher risks of COVID-19, and 85% thought that they were more likely to have COVID-19 complications (Supplementary Table 2; Table 3, A2, A14, C4, C16). This contrasted with the small number of CF-patients who were infected, as only 74 among those responding to the questionnaire had symptoms suggestive of COVID-19 (10%), and among the 20 who were tested, only one was positive. Individuals with CF were less worried about the risks from their treatment, as only 9.5 and 14% considered that CF medications were putting them at greater risk of COVID-19 or COVID-19 complications, respectively (Supplementary Table 2). Unexpectedly, CF-patients living in the Southwest of France (where low case numbers were reported at the time of the first wave) were significantly more likely to be very concerned about becoming ill with SARS-CoV-2 infection compared to those living in the Northeast of France (with high prevalence of SARS-CoV-2 cases at the time of the first lockdown) (Figure 1). On a constraint scale from 1 to 10, with 1 being “very low” and 10 being “very high,” the constraint of barrier measures was 2 [0–5 IQR] while that from the lockdown was 5 [2–8 IQR]. Results are shown for each of the French regions in Figure 1.

#### Psychological consequences of the COVID-19 pandemic and associated lockdown measures

The majority of patients (418/703; 59.5%) did not feel differently about their health status compared to 3 months earlier, whereas 161 (22.9%) felt that their health improved and 124 (17.6%) reported health degradation (Supplementary Table 2). Analysis of the 725 validated questionnaires (Table 4) showed that 43.2% of the CF-patients had signs of anxiety based on the GAD-7 questionnaire, with 25.9, 11.3, and 6.0% having mild, moderate or severe anxiety, respectively, whereas 51.0% presented signs of depression based on the PHQ-9 questionnaire, with 29.8, 14.6, 4.4, and 2.2% having mild, moderate, moderately severe and severe depression, respectively. These anxiety symptoms were also reported in interviews and were mostly related to the perceived higher risk of COVID-19 for CF-patients (Table 3, A5, A13, A15, B2). Anxiety and depression prevalence (mild, moderate or severe) was lower in the 14–18 years-old group compared to older CF-patients [anxiety: 32.5%, (14–18 years) *vs*. 43.3% (18–25 years), 48.4%, (25–35 years) and 43.0% ≥35 years; *p* = 0.049; depression: 44.7% (14–18 years) vs. 55.6% (18–25 years), 56.1% (25–35 years) and 46.6% ≥35 years; *p* = 0.066]. Furthermore, the prevalence and severity of anxiety and depression were inversely correlated with the prevalence of COVID-19 cases during the first lockdown (Figure 1). In line with the increase in anxiety and depression with age, a significant increase in smoking, consumption of alcohol and psychotic medications, and sleep disorders was found in older patients (Table 4). Assessing treatment adherence using a score of compliance/6 indicated a median [IQR] score of 1.0 (1.0–3.0) with 21% of the CF-patients having good compliance, 53% minor non-compliance and 25% non-compliance (Table 5). Interestingly, a significant difference was observed with age, with a median score of compliance/6 of 1.0 [0.0–2.0] in older patients (>35 years old) as compared with that in younger patients [1.0 (1.0–3.0) for <18, 2.0 (1.0–2.5) for 18–25 and 2.0 (1.0–3.0) for 25–35 years old; *p* = 0.0004], contrasting with the higher percentage of “good compliance” in the older group (>35 years old) with 33.5%, more than double that observed in younger patients (15.6 for <18, 15.3 for 18–25, 15.3 for 25–35 years old, *p* < 0.001) (Table 5). Moreover, non-compliance was associated with the presence of anxiety (32.8% in patients with anxiety vs. 20.1% in the non-anxious group; *p* < 0.0001) or depression symptoms (32.3% in patients with depression symptoms vs. 18.4% in the non-depressive group; *p* < 0.0001).

**Table 4:** Anxiety and depression during lockdown period.

	**Age (years)**	**[14–18]**	**[18–25]**	**[25–35]**	**≥35**	***p*-value**
	***N* = 725**	***N* = 117**	***N =* 153**	***N =* 223**	***N =* 232**	
Anxiety (GAD-7)	*N =* 717	*N =* 114	*N =* 150	*N =* 223	*N =* 230	0.09
No (0–4)	408 (56.8)	77 (67.5)	85 (56.7)	115 (51.6)	131 (57.0)	
Mild (5–9)	186 (25.9)	29 (25.4)	37 (24.7)	62 (27.8)	57 (24.8)	
Moderate (10–14)	81 (11.3)	5 (4.4)	18 (12.0)	27 (12.1)	31 (13.5)	
Severe (>15)	43 (6.0)	3 (2.6)	10 (6.7)	19 (8.5)	11 (4.8)	
Depression (PHQ-9)	*N =* 720	*N =* 114	*N =* 151	*N =* 223	*N =* 232	<0.001*
No (0–4)	352 (48.9)	63 (55.3)	67 (44.4)	98 (44.0)	124 (53.5)	
Mild (5–9)	215 (29.8)	30 (26.3)	33 (21.9)	75 (33.6)	77 (33.2)	
Moderate (10–14)	105 (14.6)	18 (15.8)	33 (21.9)	35 (15.7)	19 (8.2)	
Moderately severe (15–19)	32 (4.4)	2 (1.8)	13 (8.6)	8 (3.6)	9 (3.9)	
Severe (>20)	16 (2.2)	1 (0.9)	5 (3.3)	7 (3.1)	3 (1.3)	

Participants were asked to complete the 7-Item Generalized Anxiety Disorder Scale (GAD-7) (13) and the validated French version of 9-item Patient Health Questionnaire Depression Scale (PHQ-9) (14, 15). The total GAD-7 score ranges from 0 to 21, with a cut off of 10 indicating the presence of significant anxiety symptoms. GAD-7 scores of 5, 10, and 15 represent cut-off points for mild, moderate, and severe anxiety, respectively. The PHQ-9 score is composed of nine depressive symptom items listed in the Diagnostic and Statistical Manual of Mental Disorders-4th edition (DSMIV) for depression and ranges from 0 to 27 (14). PHQ-9 scores of 5, 10, 15, and 20 represent cut-off points for mild, moderate, moderately severe, and severe depression, respectively (14). Numbers in brackets refer to percentage. *Chi square test with Moderately severe and Severe categories combined due to small numbers.

**Table 5:** Impact of COVID-19 pandemic and associated lockdown measures on treatment, alcohol, and smoking behaviors.

			**Age (years)**				
		**Total**	**[14–18]**	**[18–25]**	**[25–35]**	**≥35**	***p*-value**
		**N = 725**	***N =* 117**	***N =* 153**	***N =* 223**	***N =* 232**	
		***N* (%)**	***N* (%)**	***N* (%)**	***N* (%)**	***N* (%)**	
Smoking behaviors	Initiate/Increase	10 (28.6)	0 (0.0)	2 (22.2)	6 (40.0)	2 (22.2)	0.94
	Stable	12 (34.3)	1 (50.0)	3 (33.3)	4 (26.7)	4 (44.4)	
	Decrease/Stop	13 (37.1)	1 (50.0)	4 (44.4)	5 (33.3)	3 (33.3)	
Alcohol behaviors	Initiate/Increase	64 (16.4)	0 (0.0)	10 (9.4)	20 (15.8)	34 (23.5)	0.04
	Stable	150 (38.4)	5 (38.5)	41 (38.7)	49 (38.6)	55 (37.9)	
	Decrease/Stop	177 (45.3)	8 (61.5)	55 (51.9)	58 (45.7)	56 (38.6)	
Psychotropic Medications	Initiate/Increase	26 (34.7)	0 (0.0)	1 (20.0)	9 (42.9)	16 (37.2)	0.02
	Stable	41 (54.7)	4 (66.7)	3 (60.0)	8 (38.1)	26 (60.5)	
	Decrease/Stop	8 (10.7)	2 (33.3)	1 (20.0)	4 (19.1)	1 (2.3)	
Sleep disorders	Initiate/Increase	254 (59.5)	27 (57.5)	61 (66.3)	81 (56.3)	85 (59.0)	0.034
	Stable	146 (34.2)	17 (36.2)	24 (26.1)	51 (35.4)	54 (37.5)	
	Decrease/Stop	27 (6.3)	3 (6.38)	7 (7.6)	12 (8.3)	5 (3.5)	
Compliance	Good compliance	147 (21.1)	17 (15.6)	23 (15.3)	33 (15.3	74 (33.5)	<0.0001
	Minor non-compliance	372 (53.5)	59 (54.1)	89 (59.3)	121 (56.0)	103 (46.6)	
	Non-compliance	177 (25.4)	33 (30.3)	38 (25.3)	62 (28.7)	44 (19.9)	

#### Social inequalities during COVID-19 pandemic and associated lockdown

Among the working population, 131 (44.9%) stopped working, 91 (31.2%) reduced their hours of work, and 70 (24.0%) worked more than before lockdown. Regarding the place of work, 176 (81.5%) worked from home, 29 (13.4%) were already home-based prior to the lockdown and 11 (5.1%) were still going to their place of work. Among the students, 192 (96.0%) remained schooled (online classes as all schools were closed in France at that moment).

Based on content analysis, differences by social categories among the 3 major themes of concern emerged from the interviews (Table 3, A–C). First, regarding the relationship to medical institutions, we observed that among the working and lower-middle social categories, very little information and guidance in relation to the COVID-19 pandemic was obtained or requested by the patients (Table 3, A1). These patients felt “lost” (Table 3, A2-3), forced to manage their care on their own, and plagued by feelings of anxiety (Table 3, A5-7). In the upper-middle and upper categories, some CF-patients did not hesitate to call their CF centre several times for information, providing reassurance (Table 3, A13). Second, regarding the relationship to sport and physical therapy (Table 3, B), CF-patients from the lower-middle category reported doing little or no sport, either before confinement or after (Table 3, B7). Not moving was perceived in this group as reinforcing the feeling of being alone and isolated (Table 3, B4). These CF-patients attested to their difficulty being away from their physiotherapists during lockdown (Table 3, B1–7). In CF-patients from more privileged categories status, the confinement was an opportunity to find new activities to practice physical exercise (Table 3, B9, B10). The third theme that emerged from the interviews related to psychosocial adjustment to COVID-19 pandemic and associated lockdown measures. CF-patients from the lowest social categories mostly over-confined themselves, with some not getting out at all during the lockdown (Table 3, C1–2, C6, C10–12). In contrast, some CF-patients belonging to the upper-middle and upper categories declared that they did not take additional precautions during the confinement (Table 3, C14, 15) but tried to meet outside and for some of them, even had get-together between neighbors (Table 3, C16).

## Discussion

In this qualitative and quantitative study, we show that the first lockdown did not significantly impact care as provided by CF centres. However, despite the low number of CF-patients diagnosed with COVID-19, they developed significant worsening symptoms of anxiety and depression during the first lockdown.

In France, as in other developed countries, healthcare systems faced two major issues. The first was the saturation of hospital infrastructures that have been largely oriented toward outpatient and day hospital settings over the last 10 years, and the physical and mental exhaustion of the healthcare workforce. The second was the reorganization of all the structures, procedures and workforce of the hospitals toward configurations almost entirely focused on caring for COVID-19 patients. In France, consultations fell by 40% among general practitioners and by 50% among specialists since the beginning of the pandemic, even when accounting for the surge of tele-consultations (19, 20). Concurrently, hospitals were asked to postpone consultations and surgeries considered to be non-urgent. In this context, one could have feared a disruption of the management of chronic diseases, such as CF, due to the delaying of care procedures and clinic visits, increasing the risk for morbidity and mortality. A study compared 2 cohorts of subjects in the hospital for management of liver disease before (December 2019 to February 2020) and during (March to May 2020) the COVID-19 pandemic in Austria (21). The authors assessed patients' perceptions on quality of care by telesurvey (cohort 1) and written questionnaire (cohort 2), but also by trends in elective and non-elective admissions. During this time, more than 90% had contact, either by telehealth or in-person visits. However, 57% reported that contacting their physician during the pandemic was difficult to impossible, and despite fewer hospital admissions, the proportion of non-elective and intensive care unit admissions increased, as did the 30-day liver-related mortality. In the present study, almost 2/3 of the consultations were maintained or replaced by telehealth and almost all necessary hospitalizations occurred (mostly in their affiliated centers). Consultations for physiotherapy were more affected as they were canceled by physiotherapist or CF-patients. However, self-drainage was used as a substitute as it is a common practice in CF. Finally, intravenous antibiotic treatments were mostly performed at home without delay, which is also a common practice in CF-patients. This low impact of the first wave of the pandemic on CF care in France, but also in other countries with similar organization, is likely due to the availability of CF Centres that were able to adjust and initiate rapid changes in care delivery (4, 22). This may have facilitated the preservation of usual care for most CF-patients, even though adult pulmonary specialists were not readily available due to their taking care of COVID-19 patients.

In the absence of available vaccines, social distancing is one of the main tools for preventing the transmission of SARS-CoV-2. As CF increases the risk of viral and bacterial respiratory infections, CF-patients are acutely aware of the benefits of practicing social distancing (23, 24). This can explain that constraint from this barrier measure was reported to be low in our study. In contrast, more patients were affected by the restrictions from the lockdown.

One common observation in patients with chronic disease was an increase in anxiety and depression symptoms. In the general population, results from surveys conducted early in the COVID-19 pandemic (March–June 2020) have shown increased prevalence of mental health symptoms, especially among young adults (25–29). People with CF are two to three times more likely to experience depression, anxiety, or both, compared to people in the general population (18, 30, 31). In France, data from the CF registry identified about 8% of patients with depression symptoms (diagnosed or followed), increasing with age, from 4% in adolescents to 16% in subjects older than 35 years old (2). The results of our study suggest that anxiety and depression symptoms were significantly increased in CF-patients during the first lockdown in France, and an increase with age was also observed. This resulted in an increase in sleep disorders, together with increased alcohol and psychotropic medication use. Surprisingly, anxiety and depression symptoms were more frequent in geographic areas where COVID-19 prevalence was low, while they were less important in areas with higher prevalence, suggesting that these symptoms do not correlate with the real risk of disease in this population.

This discrepancy may also be explained by concern about the impact of COVID-19 on the healthcare system. Blendon et al. (32) undertook a survey of residents from Hong Kong, Taiwan, Singapore, and the United States to understand the public reaction to the use of widespread quarantine. In this survey, 44 and 69% reported that they were very worried about not being able to get the healthcare or prescription drugs they needed during the quarantine period, respectively.

The impact of COVID-19 and associated measures on mental disorders during the lockdown is not limited to CF-patients. Portuguese patients with rheumatoid arthritis experienced significant worsening of their symptoms, along with anxiety and depression during the first confinement (21). Similar trends were reported in patients with COPD during lockdown with significantly intensified symptoms and disorders of mental illness such as fear, anxiety and depression (33). Interestingly, this was associated with increased adherence to using their preventive inhalers in more than one fourth of the subjects (34). Poor treatment adherence is common in CF (35, 36) and is likely to be worsened by decrease access to healthcare caregivers/structures, increased fears of SARS-CoV-2 contamination when having to get medications in pharmacies or increased anxiety/depression symptoms. Smith et al. (31) reported that depression symptoms in children were significantly associated with lower rates of adherence to airway clearance, whereas there was no significant association between patient's adherence to medical treatment/airway clearance and the PHQ-9 or GAD-7 scores of patients in the Turkish study. In our study, the presence of anxiety/depression symptoms during lockdown was associated with poor treatment adherence. A trend similar to that of COPD (disease with higher median age) was observed with only 20% non-compliance; however, this did not seem to be due to increased anxiety or depressive symptoms.

Several social science studies have shown that the health crisis caused by the COVID-19 pandemic is compounded by a major social crisis, having accentuated the inequalities between all social category, gender and generation (37–39).

Explanations are multifactorial, including high frequency of chronic diseases (comorbidity being critical in case of COVID-19), type of employment (service jobs, precarious jobs), impossibility of telecommuting or crowded housing (40). During the SARS-CoV-2 pandemic, it has been emphasized that the most precarious populations were more affected by COVID-19, notably because they accumulate several factors that cause the spread of the virus (41). Among them, three factors, often cumulative, reinforce social inequalities in healthcare: differences in access to care, inequalities in exposure risk and differences in vulnerability to disease (42). Interestingly, these factors were of moderate impact in our study across social categories. The very tight relationship between CF-patients and their CF healthcare team at the center may have helped maintain good level of care independently of social disparities, as there are no charges for the patient, even during the health crisis. In terms of access to care, according to the French Health Insurance registry, practice visits declined by 40% and specialist visits by 50% in the general population, while a significant decrease in access to care for CF-patients was not observed, even in the context of precarity.

The present study has several limitations. Subjects responding on a voluntary basis could limit the external validity, there were only self-reported measures with no objective measures of disease management. The questionnaires were available online with a generic link, and we could not verify that all responders were CF-patients > 14 years old. However, an information letter was provided with the questionnaire, explaining the selection criteria, and requiring for the responders to enter their age on the questionnaire, which prevented those under 14 to fill out the remaining questionnaire. Even though the number of patients included was below expectations, as the observed health access was better than expected, the precision of the estimation was good and the study powered enough to assess other outcomes and evaluate associations.

Our study has also some strengths. This was a national multicentre study with participating patients from all CF centres in France (only Corse region was underrepresented because of the absence of CF centre). The short duration of the recruitment period avoided bias in the context of a very rapidly evolving pandemic situation. Furthermore, the study used both quantitative and qualitative methodologies, which helped understanding all aspects of the COVID-19 pandemic and the resulting lockdown.

In conclusion, COVID-19 pandemic and associated lockdown measures in France had only minimal impact on the care management of CF-patients. The adaptability of well-structured CF centres in establishing short-term measures during the pandemic may be a model for the development of similar organization for other chronic lung diseases. However, the higher risk for anxiety and depression in CF-patients reported here needs to be considered when developing new models for care during future sanitary crisis.

## Data availability statement

The original contributions presented in the study are included in the article/Supplementary materials, further inquiries can be directed to the corresponding author/s.

## Ethics statement

This study was carried out in accordance with the Declaration of Helsinki, good clinical practice, and French legislation on clinical research. The study was approved by the Ethics Committee [Comité de Protection des Personnes (CPP) Est, 15/05/2020] (CPP N°20.05.11). Informed consent was obtained from all patients > 18 years old and from parents for patients <18 prior to inclusion. ClinicalTrials.gov Identifier: NCT04463628.

## Author contributions

NO performed the statistical analysis, wrote the original draft of the manuscript, and has verified all data. TP wrote and reviewed the manuscript and participated to the quantitative interviews and analysis. CD designed the study, wrote and reviewed the manuscript, and has verified all data. NR, BD, DG, HC, GT, and VP-E designed the questionnaire and reviewed the manuscript. HM, DA, CF, LC, and M-PM performed and analyzed the quantitative interviews. CJ and LA designed the statistical analysis strategy and scripts for the statistical analyses. BM, SL, A-CB, and RE conceived and designed the study, reviewed the manuscript, and verified all data. RE is the principal investigator of the MUCONFIN study, study guarantor and attests that all listed authors meet authorship criteria, and that no others meeting the criteria have been omitted. All authors contributed to the article and approved the submitted version.

## Funding

This study was performed under the frame of the REMEDIA project funded by the European Union's Horizon 2020 Research and Innovation Program under grant no: 874753.

## Conflict of interest

The authors declare that the research was conducted in the absence of any commercial or financial relationships that could be construed as a potential conflict of interest.

## Publisher's note

All claims expressed in this article are solely those of the authors and do not necessarily represent those of their affiliated organizations, or those of the publisher, the editors and the reviewers. Any product that may be evaluated in this article, or claim that may be made by its manufacturer, is not guaranteed or endorsed by the publisher.

## Supplementary material

Supplementary Figure 1 Flow chart of the study.

Supplementary Figure 2 Number of patients hospitalized for COVID-19 on May 24th, 2020 in the different geographic regions of France.

Supplementary Table 1 Repartition of CF patients among the different geographic metropolitan regions of France.

Supplementary Table 2 COVID-19 disease and beliefs about the risk of COVID-19 infection.
