# Supplementary figures and images for: Impact of the COVID-19 pandemic and associated lockdown measures on the management, health, and behavior of the cystic fibrosis population in France during 2020 (MUCONFIN)
Source: Front Public Health. 2022 Nov 14;10:978627. doi: 10.3389/fpubh.2022.978627 (PMC9703073; doi:10.3389/fpubh.2022.978627)

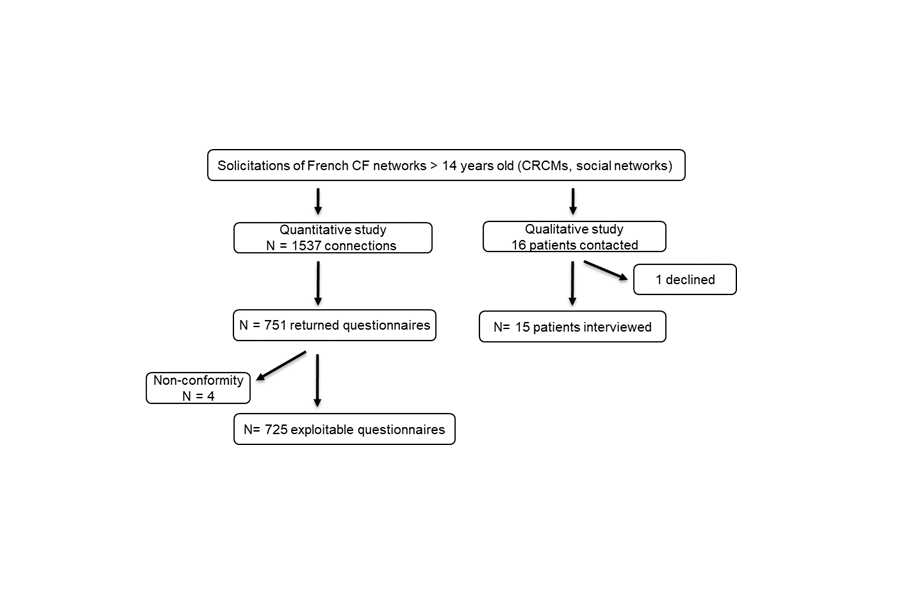

Supplement: Supplementary Figure 1 — Flow chart of the study. [file Image_1.TIF]

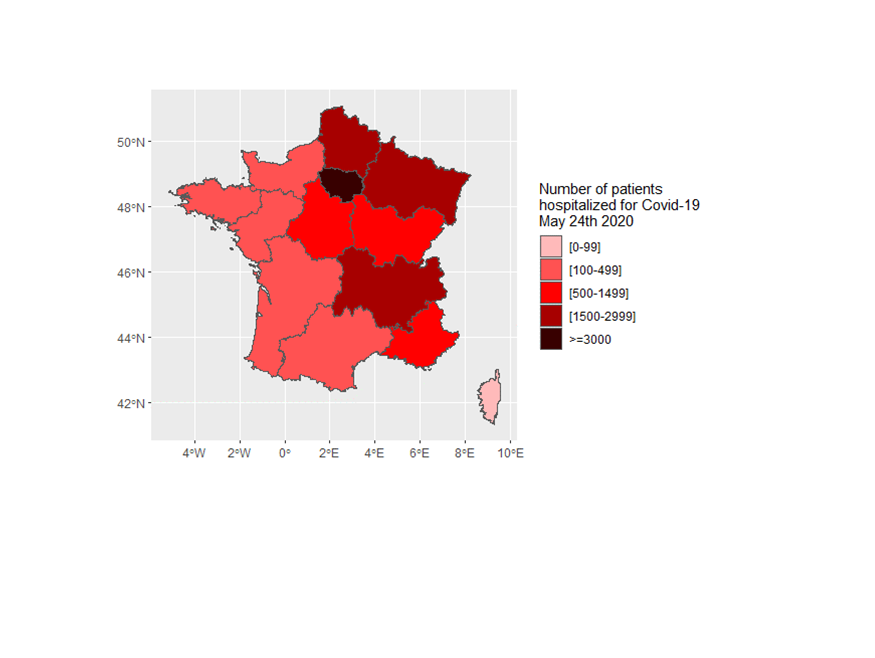

Supplement: Supplementary Figure 2 — Number of patients hospitalized for COVID-19 on May 24th, 2020 in the different geographic regions of France. [file Image_2.TIF]
