# Supplementary material for: Impact of the COVID-19 pandemic and associated lockdown measures on the management, health, and behavior of the cystic fibrosis population in France during 2020 (MUCONFIN)
Source: Front Public Health. 2022 Nov 14;10:978627. doi: 10.3389/fpubh.2022.978627 (PMC9703073; doi:10.3389/fpubh.2022.978627)
Supplement: Supplementary Table 1 — Repartition of CF patients among the different geographic metropolitan regions of France. [file Data_Sheet_1.DOCX]

Highlights

*Evidence before this study*

While the COVID-19 pandemic progressively spread across all metropolitan regions of France, most of the studies on cystic fibrosis (CF) focused on SARS-CoV-2 prevalence and suggested a low incidence of infection in this population. However, the possible indirect impact of the lockdown and barrier measures on patient care, health, and behaviours had not yet been studied in CF.

*Added value of this study*

We performed a multicentre quantitative study of the CF French population through a national online questionnaire and a qualitative study on a representative sample of CF patients. The timeframe of the study was set to precisely assess the impact of the first lockdown as close as possible to its end. This approach allowed us to obtain novel quantitative observations about the effects of the lockdown on access to care and changed behaviours in a large and diverse pool of CF patients. We also explored the impact of lockdown on mental health, which had been poorly explored thus far in the CF population.

*Implications of all the available evidence*

The present study show that the first confinement had actually only little impact on the care of CF-patients in France, with the exception of physiotherapy. These results highlight the benefits of developing specialized centres dedicated to the care of chronic and/or rare lung diseases such as CF. The adaptability of these dedicated centres contributes to improve the management during a sanitary crisis such as the COVID-19 pandemic. However, the higher risk for anxiety and depression in CF-patients reported here, independently of SARS-CoV-2 prevalence, highlights that rapid changes in care protocols should be carefully introduced, as they may affect the in-person contact aspects of patient care (physiotherapy for example) along with their physical and mental health. It would be of interest to determine if similar results are found during subsequent lockdowns, as experienced in the continuing the pandemic.
