# Supplementary material for: Impact of the COVID-19 pandemic and associated lockdown measures on the management, health, and behavior of the cystic fibrosis population in France during 2020 (MUCONFIN)
Source: Front Public Health. 2022 Nov 14;10:978627. doi: 10.3389/fpubh.2022.978627 (PMC9703073; doi:10.3389/fpubh.2022.978627)
Supplement: Supplementary Table 2 — COVID-19 disease and beliefs about the risk of COVID-19 infection. [file Data_Sheet_2.PDF]

Table S1: repartition of CF patients among the different geographic metropolitan regions of France

	Number of CF-patients (%)
	Total (N=725)
**Auvergne-Rhône-Alpes**	140 (19.72)
**Bourgogne-Franche-Comté**	36 (5.07)
**Bretagne**	58 (8.17)
**Centre-Val de Loire**	33 (4.65)
**Corse**	5 (0.70)
**Grand Est**	49 (6.90)
**Hauts-de-France**	34 (4.79)
**Normandie**	45 (6.34)
**Nouvelle-Aquitaine**	42 (5.92)
**Occitanie**	82 (11.55)
**Pays de la Loire**	30 (4.23)
**Provence-Alpes-Côte d'Azur**	85 (11.97)
**Île-de-France**	71 (10.00)

Table S2: COVID-19 disease and beliefs about the risk of Covid-19 infection.

		No. (%)
**Covid status**		
Symptoms suggestive of COVID-19		74 (10.3)
Tested for COVID-19		20 (27.4)
Positive test for COVID-19		19 (95.0)
Household diagnosed with COVID-19		12 (1.7)
**Fear of COVID-19**		
CF puts you at greater risk of COVID-19	yes	466 (64.7)
	no	176 (24.4)
	?	78 (10.8)
CF puts you at greater risk of COVID-19 complications	yes	612 (85.0)
	no	44 (6.1)
	?	64 (8.9)
Your CF medications put you at greater risk of COVID-19	yes	63 (9.5)
	no	423 (63.9)
	?	176 (26.6)
Your CF medications put you at greater risk of COVID-19 complications	yes	93 (14.1)
	no	350 (52.9)
	?	219 (33.1)

Table S2: questionnaire (in French)

Questionnaire MUCONFIN

1. Questions générales :
2. Accès aux soins Pendant le confinement
3. Anxiété et Dépression (GAD-7, PHQ-9)
4. Tabac, Alcool, drogues et Psychotrope
5. Observance
6. Crainte par rapport au Covid
7. Connaissance de l’infection à coronavirus
8. Contrainte liée aux mesures de confinement
9. Comment va ma maladie

Générale :

1. **Êtes-vous :**
    1. Un homme
    2. Une femme

2. Quel âge avez-vous ?

/__/__/ ans

3. Merci d’indiquer le code postal de votre commune

/__/__/__/__/__/

4. En ce moment, quelle est votre activité principale ?

1. Salarié du secteur privé
2. Salarié d'une entreprise publique ou nationale
3. Salarié du secteur public
4. A votre compte
5. A la recherche d'un premier emploi
6. A la recherche d'un emploi (vous avez déjà travaillé)
7. A la retraite
8. Au foyer
9. Elève ou étudiant
10. Dans une autre situation

5. En ce moment, de combien de personnes se compose votre foyer, vous y compris ?

/__ /__/ personnes

6. Êtes-vous toujours étudiant ?

Oui

Non

6. Si oui, quel est votre niveau d'étude en ce moment ?

- 6ème
- 5ème
- 4ème
- 3ème
- 2nde

- Première
- Terminale
- Université
- Autre :

Accès aux soins Pendant le confinement

7. Pendant le confinement, avez-vous eu une ou des :

	Annulé par vous	Annulé par l'hôpital	Si annulé téléconsultation proposée	Si annulé téléconsultation acceptée	Reportée	Maintenue	Non prévue
Consultations médicales à l'hôpital	1☐	2☐	3☐	4☐	5☐	6☐	7☐
Consultations médicales en ville	1☐	2☐	3☐	4☐	5☐	6☐	7☐
Consultations diététiques ?	1☐	2☐	3☐	4☐	5☐	6☐	7☐
Bilan annuel ?	1☐	2☐	3☐	4☐	5☐	6☐	7☐

	Annulé par vous	Annulé par l'hôpital	Si annulé programme web proposé	Si annulé téléconsultation	Reportée	Maintenue	Non prévue
Consultations kinésithérapeute ?	1☐	2☐	3☐	4☐	4☐	4☐	4☐

	Remplacée par HAD	Dans un autre hôpital qu'habituellement	Reportée	Oui	Non
Hospitalisation(s) pendant le confinement ?	1☐	2☐	3☐	4☐	4☐

	Faite à la maison	Remplacé par une cure antibiotique Oraux	Reportée	Oui	Non
Cure(s) antibiotique intra-veineuse(s) ?	1☐	2☐	3☐	4☐	4☐

Anxiété et Dépression (GAD-7, PHQ-9)

**8. Au cours des 2 dernières semaines, selon quelle fréquence avez-vous été gêné(e) par les problèmes suivants ?** *(Veuillez cocher (✓) votre réponse)*	Plusieurs jours	Plus de la moitié du temps	Presque tous les jours
**1.** Un sentiment de nervosité, d'anxiété ou de tension	1	2	3
**2.** Une incapacité à arrêter de s'inquiéter ou à contrôler ses inquiétudes	1	2	3
**3.** Une inquiétude excessive à propos de différentes choses	1	2	3
**4.** Des difficultés à se détendre	1	2	3
**5.** Une agitation telle qu'il est difficile à tenir en place	1	2	3
**6.** Une tendance à être facilement contrarié(e) ou irritable	1	2	3

7. Un sentiment de peur comme si quelque chose de 0 1 2 3 terrible risquait de se produire

9. **Au cours des Au cours des 2 dernières semaines, selon quelle fréquence avez-vous été gêné(e) par les problèmes suivants ?**

(Veuillez cocher (✓) votre réponse)	**Jamais**	**Plusieurs jours**	**Plus de la moitié du temps**	**Presque tous les jours**
**1.** Peu d'intérêt ou de plaisir à faire les choses	0	1	2	3
**2.** Être triste, déprimé(e) ou désespéré(e)	0	1	2	3
**3.** Difficultés à s'endormir ou à rester endormi(e), ou dormir trop	0	1	2	3
**4.** Se sentir fatigué(e) ou manquer d'énergie	0	1	2	3
**5.** Avoir peu d'appétit ou manger trop	0	1	2	3
**6.** Avoir une mauvaise opinion de soi-même, ou avoir le sentiment d'être nul(le), ou d'avoir déçu sa famille ou s'être déçu(e) soi-même	0	1	2	3
**7.** Avoir du mal à se concentrer, par exemple, pour lire le journal ou regarder la télévision	0	1	2	3
**8.** Bouger ou parler si lentement que les autres auraient pu le remarquer. Ou au contraire, être si agité(e) que vous avez eu du mal à tenir en place par rapport à d'habitude	0	1	2	3
**9.** Penser qu'il vaudrait mieux mourir ou envisager de vous faire du mal d'une manière ou d'une autre	0	1	2	3

10. **Si vous avez coché au moins un des problèmes évoqués, à quel point ce(s) problème(s) a-t-il (ontils) rendu votre travail, vos tâches à la maison ou votre capacité à vous entendre avec les autres difficile(s) ?**

Pas du tout difficile(s)	Assez difficile(s)	Très difficile(s)	Extrêmement difficile(s)
⑤	⑤	⑤	⑤

Tabac, Alcool et Psychotrope

11. Actuellement, est-ce que vous fumez ?

1. Oui, des cigarettes, y compris des cigarettes roulées
2. Uniquement d'autres types de tabac (pipe, cigare, chicha…)
3. Non, je ne fume pas

12. Si fumeur

Tab2. Par rapport à avant les mesures de confinement, comment a évolué votre consommation de tabac ?
1. Elle a augmenté
2. Elle est restée stable
3. Elle a diminué

13. Habituellement, est-ce que vous buvez ?

1. Oui régulièrement
2. Oui, mais seulement en compagnie d'autres personnes
3. Non, je ne bois pas

14. Par rapport à avant les mesures de confinement, comment a évolué votre consommation de boissons alcoolisées, qu'il s'agisse de bière, de vin, de cidre, d'alcool fort, de champagne ou tout autre type d'alcool même peu alcoolisé ?

1. Elle a augmenté
2. Elle est restée stable
3. Elle a diminué
4. Je ne consomme jamais d'alcool

15. Actuellement, est-ce que vous prenez des drogues non-autorisées (marijuana, cocaïnes…) ?

1. Oui

3. Non, je n'en prends pas

Si oui :

16. Par rapport à avant les mesures de confinement, comment a évolué votre consommation

1. Elle a augmenté
2. Elle est restée stable
3. Elle a diminué

17. Actuellement, est-ce que vous prenez des psychotropes (somnifères, autres…) ?

1. Oui
3. Non, je n'en prends pas

Si oui

18. Par rapport à avant les mesures de confinement, comment a évolué votre consommation

1. Elle a augmenté
2. Elle est restée stable
3. Elle a diminué

19. **Actuellement, est-ce que vous avez des troubles du sommeil (difficultés d'endormissement, réveil)** ?

1. Oui
3. Non, je n'en ais pas

Si oui

20. Par rapport à avant les mesures de confinement, comment a évolué votre trouble du sommeil

1. ils ont augmenté
2. Ils sont restés stable
3. Ils ont diminué

Observance

21. **Ce matin, avez-vous omis de prendre votre traitement ?**

Oui

Non

22. **Depuis la dernière visite, avez-vous été à court de traitement ?**

Oui

Non

23. Avez-vous déjà pris votre traitement avec un retard par rapport au calendrier habituel ?

Oui

Non

24. Avez-vous déjà manqué votre traitement à cause d'un oubli ?

Oui

Non

25. Avez-vous déjà décidé d'arrêter un traitement en raison d'effets secondaires ?

Oui

Non

26. Pensez-vous que le nombre de comprimés que vous devez prendre chaque jour est trop élevé ?

Oui

Non

Crainte par rapport au Covid

27. Pensez-vous que votre maladie chronique la mucoviscidose vous rend plus à risque d'une infection à Coronavirus?

Oui
Non

28. **Pensez-vous que votre maladie chronique la mucoviscidose vous rend plus à risque de complications en cas d'infection à coronavirus ?**

Oui
Non

29. **Prenez-vous un des médicaments suivants sur une base régulière?**

Cortisone (Corticoïdes par la bouche)
Cortisone (Corticoïdes par voie respiratoire)
AINS ,
Zithromax,
Xolair ou autres Biothérapie
Aérosol (antibiotique ou autre)
Lavage de nez
Je ne prends aucun de ces médicaments
Autre :

30. Pensez-vous que vos médicaments vous mettent plus à risque d'infection au COVID-19?

Oui
Non
Je ne sais pas
Je ne prends aucun médicament.

31. Pensez-vous que vos médicaments vous mettraient plus à risque de complications en cas d'infection au COVID-19?

Oui
Non
Je ne sais pas
Je ne prends aucun médicament.

Connaissance de l'infection à coronavirus

32. Pensez-vous être bien informée par rapport au COVID-19?

Oui
Non

33. Quelles sont vos sources d'information ?

- Organisations de santé (OMS-Organisation Mondial de la Santé, Ministère de la santé, sites internet des hôpitaux, etc.)
- Professionnels de la santé (Médecins, infirmiers, etc.)
- Membres de famille et amis
- Radio ou télévision
- Conférences de presse quotidiennes gouvernementales
- Autre sources gouvernementales
- Informations communiquées par l'école ou l'université
- Réseaux sociaux (whatsapp, facebook, intagram, ….)

34. Vrai ou Faux (lisez attentivement les questions)

- COVID-19 est une maladie respiratoire infectieuse causée par une bactérie.
- COVID-19 survient indépendamment du pays ou du groupe ethnique.
- Les symptômes apparaissent habituellement entre 2 et 14 jours après l'exposition à une personne ou une surface contaminée.
- COVID-19 peut être transmis de personne à personne par des gouttelettes de salive ou d'éternuement.
- COVID-19 ne peut pas être transmis en touchant quelque chose ou quelqu'un contaminé, puis en touchant la bouche, le nez ou les yeux.
- Se laver les mains vigoureusement (avec de l'eau et du savon pendant au moins 20 secondes) aide dans la prévention de la transmission.
- L'utilisation d'une solution hydro-alcoolique pour le lavage des mains aide dans la prévention de la transmission.
- Lorsqu'une personne tousse fréquemment, l'utilisation d'un masque peut réduire la transmission par gouttelette de COVID-19.
- COVID-19 est seulement retrouvé chez des individus qui ont des signes ou des symptômes.
- COVID-19 est seulement dangereux pour les personnes âgées.

35. Les symptômes suivants peuvent-ils être associés à une infection de COVID-19? Vrai/ Faux (ou coché)

- Diarrhées

- Difficulté à respirer
- Essoufflement
- Fatigue
- Fièvre
- Mal au ventre
- Boutons sur la peau
- Toux sèche
- Vomissements
- Perte de l'odorat

36. Avez-vous eu des symptômes faisant évoquer une infection à Coronavirus ?

1. Oui
2. Non

37. Vous a-t-on fait un test pour savoir si c'était le Coronavirus (COVID-19) ?

3. Oui
4. Non

38. Quel a été le résultat de ce test ?

1. Il a confirmé que c'était le Coronavirus (COVID-19)
2. Il a indiqué que ce n'était pas le Coronavirus (COVID-19)

39. Depuis l'annonce de fermeture des écoles/universités/ lieux de travail par le gouvernement, j'évite les transports en commun.

Oui
Non
Ne s'applique pas

40. Depuis l'annonce de la fermeture des écoles/universités par le gouvernement OU depuis votre confinement...

(Échelle Jamais, Rarement, Parfois, Souvent, Toujours)

- J'ai annulé ou déplacé des rencontres avec des amis, des sorties aux restaurants, ou des événements sportifs.
- Je nettoie et je désinfecte les objets qui sont souvent touchés par les mains (ex: poignée de porte, surfaces)
- Je lave mes mains pour la prévention de COVID-19
- Autant que possible, j'évite de tousser quand il y a des gens proches de moi.
- J'ai discuté avec ma famille, mes amis ou des professionnels de santé des mesures préventives à prendre.
- J'ai discuté avec ma famille, mes amis ou des professionnels de santé des gestes à poser si on devient infecté.
- J'évite les places avec beaucoup de personnes (places publiques, supermarché, etc.)

41. Je pense que COVID-19 est dangereux pour moi.

(Echelle Pas du tout d'accord - 0-10 - Tout à fait d'accord)
1. Je pense que le COVID-19 est dangereux pour mes proches.
(Echelle Pas du tout d'accord - 0-10 - Tout à fait d'accord)

2. J'ai peur d'être infecté.e par le COVID-19.

(Echelle Pas du tout d'accord - 0-10 - Tout à fait d'accord)

3. J’ai peur que les membres de ma famille soient infectés par le COVID-19.

(Echelle Pas du tout d'accord - 0-10 - Tout à fait d'accord)

4. Mes parents sont inquiets par rapport au COVID-19.

(Echelle Pas du tout d'accord - 0-10 - Tout à fait d'accord)

Contrainte liée aux **mesures** de confinement

42. Les mesures d’hygiène renforcée recommandées par les pouvoirs publics pour lutter contre le Coronavirus (COVID-19) sont-elles contraignantes pour vous ?
(Mesures d’hygiène renforcée comme se laver très régulièrement les mains, tousser ou éternuer dans son coude ou dans un mouchoir, utiliser un mouchoir à usage unique et le jeter…)

Donnez une note entre 0 et 10 : la note 0 indique que ces mesures ne sont pas du tout contraignantes pour vous et la note 10 que ces mesures sont très contraignantes pour vous.
Les notes intermédiaires permettent de nuancer votre jugement.

0	1	2	3	4	5	6	7	8	9	10

Je ne sais pas

43. Les mesures de confinement imposées par les pouvoirs publics pour lutter contre le Coronavirus (COVID-19) sont-elles contraignantes pour vous ?
(Mesures de confinement comme la limitation de la circulation des personnes, le confinement au domicile de tous les Français qui peuvent rester chez eux, la fermeture des lieux publics non indispensables, des écoles, des garderies et des universités, l’interdiction des rassemblements publics**…)**

Donnez une note entre 0 et 10 : la note 0 indique que ces mesures ne sont pas du tout contraignantes pour vous et la note 10 que ces mesures sont très contraignantes pour vous.
Les notes intermédiaires permettent de nuancer votre jugement.

0	1	2	3	4	5	6	7	8	9	10

Je ne sais pas

44. Quelles sont vos activités depuis votre confinement ?
Activités de loisir (broderie, cuisine, jouer de la musique, etc.)
Commencer/continuer un projet personnel (chaîne Youtube, blog personnel, etc.)
Suivre des classes en ligne sur des sujets qui m’intéressent
Divertissement (jeux vidéo, films, série télé, etc.)
Étude scolaire
Exercices physiques
Lire des livres
Méditation
Passer du temps avec ma famille
Pratiques religieuses
Utiliser les médias sociaux
Autre :

45. Avez-vous un emploi (Temps plein ou temps partiel) ?
Oui
Non

46. Si oui, pendant la pandémie à COVID-19... ?

- Je continue à travailler comme avant
- J’ai réduit le nombre d’heures de travail
- J'ai opté pour faire du télétravail
- J’ai arrêté de travailler
- Je travaille plus qu'avant

Comment va ma maladie :

47. **Veuillez indiquer comment vous vous êtes senti(e), depuis le début du confinement :**

Avez-vous été été gêné(e) : <u>Cocher la case de votre choix</u>

	Très gêné(e)	Assez gêné(e)	Un peu gêné(e)	Pas du tout gêné(e)
**1-** Pour faire des efforts physiques importants tels que courir, faire du sport	1☐	2☐	3☐	4☐
**3-** Pour porter, soulever des choses lourdes (livres, sac de provisions, cartable...)	1☐	2☐	3☐	4☐
**4-** Pour monter un étage ....….....	1☐	2☐	3☐	4☐
**5-** Pour monter les escaliers aussi vite que les autres……………………………………………	1☐	2☐	3☐	4☐

48. Depuis le début du confinement, indiquez si confinement indiquez si :

Cocher la case de votre choix

	Tout le temps)	Souvent	Quelquefois	Jamais
**6-** Vous vous êtes senti(e) en pleine forme................	1☐	2☐	3☐	4☐
**7-** Vous vous êtes fait du souci………………………	1☐	2☐	3☐	4☐
**8-** Vous vous êtes senti(e) inutile...............................	1☐	2☐	3☐	4☐
**9-** Vous vous êtes senti(e) fatigué(e)..........................	1☐	2☐	3☐	4☐
**10-** Vous vous êtes senti(e) plein(e) d'énergie...........	1☐	2☐	3☐	4☐
**11-** Vous vous êtes senti(e) épuisé(e)..........................	1☐	2☐	3☐	4☐
**12-** Vous vous êtes senti(e) triste..........……………....	1☐	2☐	3☐	4☐

En pensant à votre état de santé depuis le début du confinement, indiquez :

Entourer la réponse de votre choix

(une seule réponse)

49. **Dans quelle mesure les repas sont un problème pour vous :**
    - Dès qu'on parle de manger, ça vous dégoûte 1
    - Vous ne mangez jamais avec plaisir 2
    - Il vous arrive de manger avec plaisir... 3
    - C'est un plaisir pour vous de manger 4

Entourer la réponse de votre choix (une seule réponse)

50. Dans quelle mesure vos traitements (y compris la kiné / autodrainage et les aérosols) vous gênent-ils dans la vie quotidienne :

- Pas du tout / un peu 1
- Modérément 2
- Beaucoup 3
- Enormément 4

Entourer la réponse de votre choix (une seule réponse)

51. Par rapport à l'année dernière à la même époque, passez-vous pour votre traitement :

- Beaucoup plus de temps 1
- Un peu plus de temps 2
- Le même temps 3
- Moins de temps 4

En pensant à votre état de santé depuis le début du confinement **, indiquez :**

Entourer la réponse de votre choix (une seule réponse)

52. **Trouvez-vous que votre santé est actuellement :**

- Très bonne 1
- Plutôt bonne 2
- Plutôt mauvaise 3
- Très mauvaise 4

53. Par rapport à il y a trois mois, que pensez-vous de votre état de santé :

Entourer la réponse de votre choix (une seule réponse)

- Vous avez le sentiment que votre état de santé s'est amélioré 1
- Vous avez le sentiment que votre état de santé s'est stabilisé 2
- Vous avez le sentiment que votre état de santé s'est dégradé 3

54. En pensant à votre état de santé depuis le début du confinement, indiquez pour chacune des phrases suivantes dans quelle mesure elles sont vraies ou fausses pour vous, en cochant la case appropriée :

	**Totalement vrai**	**Plutôt vrai**	**Plutôt faux**	**Totalement faux**
**1-** Quand je fais un effort physique, j'ai du mal à récupérer après...				
**2-** Je dois réduire les efforts physiques importants comme courir, faire du sport.............................				
**3-** Je me force à manger......................................				
**4-** Je reste souvent à lire ou devant la télévision...				
**5-** Je me trouve trop mince………………………				
**6-** Je me sens différent(e) des autres physiquement				
**7-** Je ne me sens pas sûr(e) de moi à cause de mon apparence physique……………………………				
**8-** Les gens posent des questions gênantes............				
**9-** Les gens ont peur que je sois contagieux(se).....				
**10-** Je sens que ma toux dérange les autres.............				

55. Veuillez indiquer comment vous vous êtes senti(e), depuis le début du confinement, en cochant la case appropriée.

	Beaucoup	Assez	Un peu	Pas du tout
**1. Avez-vous eu du mal à maintenir votre poids ?**	1☐	2☐	3☐	4☐
**2. Avez-vous été encombré(e) ?**	1☐	2☐	3☐	4☐
**3. Avez-vous toussé pendant la journée ?**	1☐	2☐	3☐	4☐
**4. Avez-vous eu des crachats ?**	1☐	2☐	3☐	4☐

5. Vos crachats ont-ils été plutôt :

1☐ Transparents 2☐ Clairs à jaunâtres 3☐ Foncés à verdâtres 4☐ Avec du sang

	Tous les jours	Souvent	De temps en temps	Jamais
**6. Avez-vous eu des sifflements ?**	1☐	2☐	3☐	4☐
**7. Avez-vous eu du mal à respirer ?**	1☐	2☐	3☐	4☐
**8. Avez-vous été réveillé(e) par la toux la nuit ?**	1☐	2☐	3☐	4☐
**9. Avez-vous eu de la diarrhée ?**	1☐	2☐	3☐	4☐
**49. Avez-vous eu mal au ventre ?**	1☐	2☐	3☐	4☐
